# Depression symptom profile in a nationally-representative cohort of older adults: Relationships with suicide-related outcomes

**DOI:** 10.1192/j.eurpsy.2025.2366

**Published:** 2025-08-26

**Authors:** I. Pérez-Diez, A. de la Torre-Luque, J. L. Ayuso-Mateos, B. Dolz del Castellar, M. Miret, J. Domènech-Abella, B. Olaya, J. Maria Haro

**Affiliations:** 1 Department of Psychiatry, Universidad Autónoma de Madrid; 2 Instituto de Salud Carlos III, Centro de Investigación Biomédica en Red de Salud Mental. CIBERSAM; 3 Department of Legal Medicine, Psychiatry and Pathology, Universidad Complutense de Madrid; 4 Instituto de Investigación Sanitaria Princesa (IIS- Princesa), Madrid; 5 Sant Joan de Déu Research Institute, Barcelona, Spain

## Abstract

**Introduction:**

Depression among older adults is a significant public health issue that often goes underrecognized, despite its profound impact on their quality of life and mental well-being. With advancing age, many individuals encounter challenges such as loneliness, chronic illness, and bereavement, which heighten their vulnerability to depressive symptoms. These symptoms are not only detrimental to overall health but are also strongly associated with an increased risk of suicidal behaviours. Although these suicidal behaviours are preventable, much research should be done to disentangle development pathways in older adults.

**Objectives:**

The aim of this research is to identify the symptomatic profiles of depression in the elderly population and to relate this profile to suicidal outcomes.

**Methods:**

We utilized data from the “*Edad con Salud”* cohort study, a longitudinal household survey conducted on a nationally representative sample in Spain (aged 18+ years). Participants who could not be personally interviewed and those under 50 years old were excluded, resulting in a final sample of 3,533 participants (54.31% female, age = 66.42(10.27)). Suicidal behaviours and depressive symptoms were assessed using the Composite International Diagnostic Interview (CIDI 3.0). A Latent Class Analysis (LCA) was performed at base level to identify participant profiles based on 8 depressive symptoms. We followed this with a survival analysis to assess differences between profiles in the progression of suicidal ideation and attempts in the different waves. Finally, Cox regression was used to examine the impact of sociodemographic and clinical covariates on suicidal behaviours.

**Results:**

The three-cluster model provided the best fit. The first class (72.52%) included most participants without depressive symptoms; the second class (13.67%) included participants with three symptoms (depressive mood, diminished interest or pleasure and fatigue); and the third class (13.81%) included participants with all eight symptoms (see Image 1). Survival analysis revealed significant differences between the groups over time for both suicidal ideation (*χ^2^*=1332, *p* < .01) and suicide attempts (*χ^2^*=215, *p* < .01), with the third group having the highest number of cases. Cox regression indicated that suicidal ideation was associated with the second (*HR* = 5.38) and third classes (*HR* = 59.8), age (*HR* = 0.97), and loneliness (*HR* = 0.91), among other variables. Suicide attempts were associated with the second (*HR* = 6.08) and third classes (*HR* = 25.3), age (*HR* = 0.92), and loneliness (*HR* = 1.02).

**Image 1:**

**Figure:**
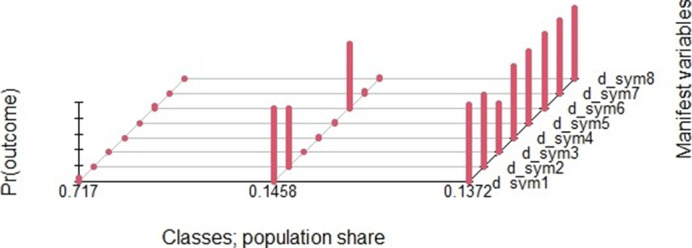

**Conclusions:**

Participants with depressive symptoms, even at a subclinical level, have an increased risk of suicidal behaviours, especially when other risk factors such as loneliness are present.

**Disclosure of Interest:**

None Declared

